# The mortality of critically ill patients was not associated with inter-hospital transfer due to a shortage of ICU beds - a single-centre retrospective analysis

**DOI:** 10.1186/s40560-020-00501-z

**Published:** 2020-10-30

**Authors:** Jonatan Oras, Marko Strube, Christian Rylander

**Affiliations:** Department of Anaesthesiology and Intensive Care Medicine, Institute of Clinical Sciences, Sahlgrenska Academy, University of Gothenburg and Sahlgrenska University Hospital, 413 45 Gothenburg, Sweden

**Keywords:** Intensive care unit, Patient transfer, Transportation of patients, Logistics, Mortality

## Abstract

**Background:**

Patients in the intensive care unit (ICU) are increasingly being transferred between ICUs due to a shortage of ICU beds, although this practice is potentially harmful. However, in tertiary units, the transfer of patients who are not in need of highly specialized care is often necessary. The aim of this study was to assess the association between a 90-day mortality and inter-hospital transfer due to a shortage of ICU beds in a tertiary centre.

**Methods:**

Data were retrieved from the local ICU database from December 2011 to September 2019. The primary analysis was a risk-adjusted logistic regression model. Secondary analyses comprised case/control (transfer/non-transfer) matching.

**Results:**

A total of 573 patients were transferred due to a shortage of ICU beds, and 8106 patients were not transferred. Crude 90-day mortality was higher in patients transferred due to a shortage of beds (189 patients (33%) vs 2188 patients (27%), *p* = 0.002). In the primary, risk-adjusted analysis, the risk of death at 90 days was similar between the groups (odds ratio 0.923, 95% confidence interval 0.75–1.14, *p* = 0.461). In the secondary analyses, a 90-day mortality was similar in transferred and non-transferred patients matched according to SAPS 3-score, age, days in the ICU and ICU diagnosis (*p* = 0.407); SOFA score on the day of discharge, ICU diagnosis and age (*p* = 0.634); or in a propensity score model (*p* = 0.229).

**Conclusion:**

Mortality at 90 days in critically ill patients treated in a tertiary centre was not affected by transfer to another intensive care units due to a shortage of beds. We found this conclusion to be valid under the assumption that patients are carefully selected and that the transports are safely performed.

**Supplementary Information:**

The online version contains supplementary material available at 10.1186/s40560-020-00501-z.

## Background

Inter-hospital transfer of critically ill patients between intensive care units (ICUs) is a common practice in Sweden [1]. This procedure is considered a means of providing best care when a patient in need of highly specialized procedures is transported to a tertiary centre. However, as elsewhere [2], patients are increasingly being transferred between ICUs due to a shortage of ICU beds. The number of ICU beds in Sweden is among the lowest in Europe, and the occupancy rate is constantly high [1, 3]. Transfer of selected ICU patients to another unit with available bed space is a frequent solution when the demand for new admittances exceeds the available capacity. Several hospitals employ dedicated ambulance systems with equipment and personnel allocated to maintain intensive care during transportation. However, there are many potentially negative effects imposed by transport that interrupts bedside care. Medical treatment is partly brought to a pause, and the patients may need intubation for safe transportation [4, 5]. Practical hazards related to the displacement itself carry risks of complications [6, 7]. The receiving ICU team may assess the patient prognosis differently and introduce restrictions to care [8, 9]. Furthermore, a recent registry analysis has shown that patients transferred due to bed shortages are exposed to a higher risk of death than that in patients who are transferred between ICUs for other reasons [10]. While it has been reported that when compared with directly admitted patients, critically ill patients referred *to* a tertiary centre have a similar adjusted mortality [11], we have found no analysis of the similarly comparative mortality of patients transferred *from* a tertiary ICU due to a limited capacity. This is an unanswered question in everyday practice in a tertiary centre where patient turnover is steadily increasing due to centralization and technical development of care. Therefore, the aim of this study was to assess the association between mortality and inter-hospital transfer from a tertiary centre ICU to other ICUs due to a shortage of ICU beds.

## Methods

This was a retrospective, a registry-based single-centre study performed at the Sahlgrenska University Hospital/Sahlgrenska, Gothenburg, Sweden. The study was approved by the Regional Research Ethics Committee of Gothenburg, Sweden, on April 6, 2017 (Approval number 223-17). Patient consent was waived due to the retrospective nature of the analysis based on existing data. The manuscript was prepared according to the Strengthening The Reporting of Observational Studies in Epidemiology (STROBE) guidelines [12].

### Setting

The Sahlgrenska University Hospital is an administrative conglomerate of four neighbouring hospitals. The major one, originally the Sahlgrenska Hospital, is the largest hospital in Sweden and a tertiary referral centre for major trauma, major vascular and upper abdominal surgery, spinal surgery, radiological interventions, hepatic failure and liver transplantation. The hospital is also a centre for coronary revascularization, embolectomy for acute stroke and haematological stem cell transplantation. Patients with unselected acute admissions are mixed with the tertiary care patient population. Critically ill patients within this case mix are treated in the Central ICU (CICU), which hosts slightly above 2000 yearly admittances.

### Reasons for outbound transfer

Due to limited bed availability, patient turnover is high in the CICU. Outbound patient transfers are an essential element of logistics. For each patient being transported to another ICU during ongoing intensive care, the reason is classified and registered according to standards in the Swedish Intensive Care Registry. Three categories are used: (1) home address within the catchment area of the receiving hospital, (2) medical (tertiary) treatment completed, and (3) shortage of ICU beds. Patients transferred for the latter reason were included in the present study.

### Patient selection for transfer

When a transfer to another ICU is necessary due to a shortage of capacity, there is a rigorous selection of patients who are expected not to be negatively affected by the transport or the change of hospital. The standard operation procedure (SOP) is based on a mutual assessment of a transferable patient by the referring and the receiving ICU, present level and components of care, future treatment and potential for recovery. The responsible persons are the heads of the respective ICU. The aim of this process is to select the candidate who is the most stable and suited for transport and does not have an imminently poor prognosis for care in the new ICU. A presumptively good matching between the patient needs and the standard of care in the ICU at the destination is facilitated by the official standards for intensive care in different hospitals [13].

### Means of transport

The patient transports are performed according to current recommendations [5]. There are specially designed ambulances allowing secure loading of the necessary equipment for ongoing intensive care, which is delivered by an ICU physician or, in some cases, by a registered nurse specialized in anaesthesia. Medical devices (ventilator, automatic syringes) and monitoring equipment (pulse-oximeter, electrocardiogram, arterial line with continuous blood pressure) are fitted to special supports, and drugs needed for possible adverse events are provided by the referring ICU.

### Patients and study variables

We used the local ICU registry to identify patients and extract data that had been validated and exported to the Swedish Intensive Care Registry. Inclusion criteria were adult patients admitted to the CICU between December 1, 2011, and September 30, 2019, with this period being defined by the introduction of the SAPS 3 score in the unit. Patients transferred due to a shortage of ICU beds were identified according to the registered reason for outbound transfer. In addition, we considered that patients discharged to another ICU within the Sahlgrenska University Hospital and patients discharged to another hospital not matching the patient’s resident postal code were transferred due to a shortage of ICU beds. When the registered reason for transfer did not match this assumption, the case was manually inspected and classified accordingly. Patients admitted for scheduled postoperative care were not included in the analysis because they normally are not eligible for transfer to other ICUs.

The following variables were obtained: patient age, patient sex, patient postal code, date and time of admission, date and time of discharge, time in the ICU, discharging destination (other hospital ICU or ward in our hospital), survival status (yes/no) and possible date of death, total Nine Equivalents of Nursing Manpower Score (NEMS), surgical status (elective surgery, acute surgery, no surgery), main ICU diagnosis (ICD code), and total SAPS 3 score. From January 1, 2017, admission, daily and discharge Sequential Organ Failure Assessment (SOFA) scores were available in the study database. For the analysis, we used the ICD-10 code of the primary ICU diagnosis (i.e., the main reason for intensive care) registered in the database. The complete list of ICD codes and the corresponding ICU diagnosis is presented in Additional file 1.

### Outcome variables and analyses

The main outcome variable was death at 90 days of patients transferred due to a shortage of ICU beds as compared to non-transferred patients. The primary analysis employed transfer status as the independent variable in a risk-adjusted logistic regression model. The risk-adjusted model was created by using a stepwise forward logistic regression model with two blocks. In the first block, SAPS 3, age, sex, surgical status and NEMS score were included. In the second block, the primary ICU diagnoses were tested. At each step, variables not in the model were tested with the variables in the model one at the time. The variable with the lowest *p* value, as tested in the model, were included in the model at each step. This was repeated until there were no remaining variables with a *p* value < 0.05. The details of this model are described in Additional data.

The secondary analyses were performed with case-control matching in three different ways:

1. Patients were matched according to their admission status with the following variables: the first three characters in the ICD code of the primary ICU diagnosis, SAPS 3 score ± 10, age ± 10 and according to the time on ward, i.e., a transported patient could only be matched with a non-transferred patient with the same or a greater number of ICU days.
2. In a subset of patients with available SOFA scores, matching was performed with the following variables: SOFA score on the day of discharge in a transferred patient (case) and a non-transferred patient with the same SOFA score ± 2 on the corresponding day (control). Patients were also matched according to primary ICU diagnosis and age (categorised as the following: 0–18 years, 18–40 years, 40–60 years, 60–70 years, 70–80 years and 80+ years).
3. Patients were matched with propensity scores. The propensity score was calculated on the probability of being transferred, taking into account the following matching variables: sex, SAPS 3 score, ICU days, mean NEMS and primary ICU diagnosis. The propensity score tolerance was set to 0.02 in the matching process.

### Statistics

Continuous variables were checked for normality. Normally distributed variables are presented as the mean ± standard deviation, and non-normally distributed variables are presented as the median (interquartile range). The *t* test was used for comparison of means on normally distributed variables, and the Mann-Whitney *U* test was used for comparison of distributions of non-normally distributed variables. Fisher’s exact test was used for comparison of binary variables with dichotomous outcomes. Mortality at 90 days was compared between the groups with univariate and multivariable logistic regression (primary analysis). The log rank test was used to compare incidences over time between the case/control groups, and unmatched patients were removed from the analysis. A *p* value < 0.05 was considered significant. IBM SPSS version 24.0 was used in the matching process and the statistical analyses. No power calculation was performed, and all data available from the introduction of the SAPS 3 score in the unit was used in the analysis.

## Results

### Study cohort

**Fig. 1:**
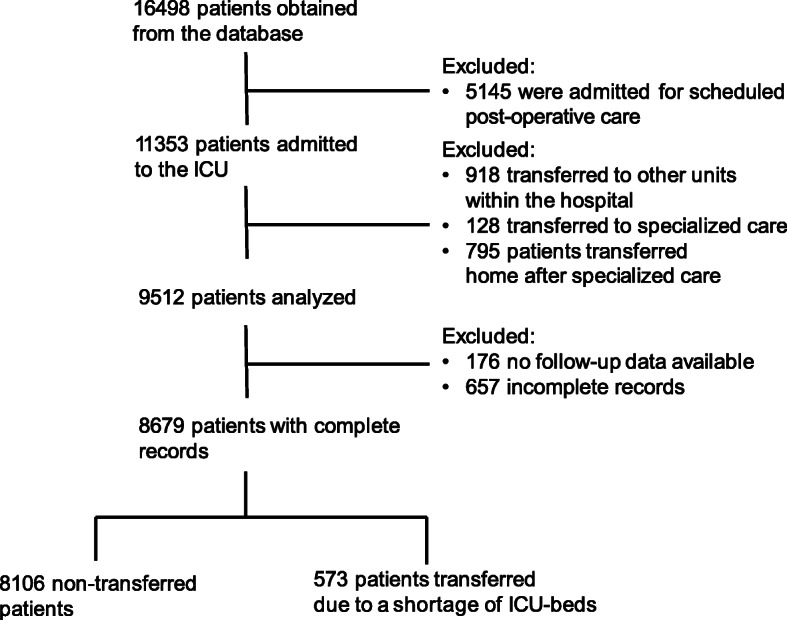
Study flow chart. ICU; Intensive care unit

A total of 16,498 patients were identified in the database. Of these, 5145 were excluded due to admission for scheduled postoperative care. Another 1841 patients were excluded due to transfer within the same hospital or to highly specialized units, e.g., a burn-centre or paediatric ICU, or transfer to their home hospital after specialized care. Next, 833 patients were excluded due to missing data, i.e., the SAPS 3 score or ICU diagnosis was not recorded, or no follow-up data were available. A total of 8679 patients were included in the final analysis. After net correction of erroneous classifications (*n* = 161), 573 patients remained in the group that was transferred due to a shortage of ICU beds (Fig. 1). The nine hospitals involved in receiving these patients had a median (interquartile range) standardised mortality rate (SMR) according to SAPS3 of 1.05 (0.99 - 1.14) during the study period [1].

### Patient characteristics

The median age within the whole cohort was 64 (48–74) years, and 3474 (40%) were women. The mean SAPS 3-score was 59 ± 19, and 3380 (39%) patients were admitted after acute or elective surgery. The most common ICU diagnosis was respiratory failure due to COPD/asthma (*n* = 800, 9%), followed by infection/sepsis (*n* = 771, 9%), cardiac arrest (*n* = 725, 8%), trauma (*n* = 675, 8%) and intoxication (*n* = 482, 6%). The median ICU stay was 2 (1–4) days, and the median NEMS was 32 (24–39) points/day.

Patients transferred due to a shortage of ICU beds were more likely to be of higher age and to have a higher SAPS 3-score, a higher mean NEMS and a longer ICU stay in our unit. They were also less likely to have been subjected to acute or elective surgery. Furthermore, they were more likely to have a diagnosis of infection/sepsis, cardiac arrest, respiratory tract infection, COPD/asthma, acute abdomen (except gastrointestinal bleeding), renal/urological disease and trauma, as well as less likely to have malignancy, subarachnoid haemorrhage, other cerebrovascular events, acute aortic rupture/dissection, peripheral artery disease, circulatory shock, liver failure, transplantation or postoperative care (Table 1).

**Table 1:** Patient characteristics

		Non-transferred patients (*n* = 8106)	Transferred due to shortage of ICU beds (*n* = 573)
Demographics	Female sex, *n* (%)	3250 (40)	224 (39)
	Age, years	63 (48–73)	66 (50–74)*
ICU data	SAPS 3 score	57 ± 16	62 ± 15***
	ICU days, days	2 (1–4)	3 (2–5)***
	Mean NEMS	32 (24–39)	38 (34–43)***
Surgery	No surgery, *n* (%)	4900 (60)	399 (70)***
	Acute surgery, *n* (%)	2176 (27)	138 (24)*
	Elective surgery, *n* (%)	1030 (13)	36 (6)***
Primary ICU diagnosis	Infection/sepsis, except respiratory tract infection, *n* (%)	674 (8)	97 (17)***
	Malignancy, *n* (%)	243 (3)	0 (0)***
	Haematological disease, *n* (%)	35 (0)	0 (0)
	Endocrinal disease, *n* (%)	289 (4)	16 (3)
	Intoxication, *n* (%)	458 (6)	24 (4)
	Neurological disease, *n* (%)	432 (5)	20 (3)
	Cardiac disease, except cardiac arrest, *n* (%)	390 (5)	19 (3)
	Cardiac arrest and asphyxia, *n* (%)	655 (8)	70 (12)*
	Subarachnoid haemorrhage, *n* (%)	78 (1)	0 (0)*
	Cerebrovascular event, except SAH, *n* (%)	231 (3)	7 (1)*
	Acute aortic rupture or dissection, *n* (%)	182 (2)	5 (1)*
	Arterial disease, not ruptured aorta, *n* (%)	251 (3)	2 (0)***
	Musculoskeletal disease, *n* (%)	6 (0)	0 (0)
	Circulatory shock, other, *n* (%)	132 (2)	3 (1)*
	Respiratory tract infection, *n* (%)	361 (4)	69 (12)***
	COPD/asthma, *n* (%)	733 (9)	67 (12)*
	Respiratory tract disease, except infection or COPD/asthma, *n* (%)	128 (2)	6 (1)
	Renal/urologic disease, *n* (%)	108 (1)	15 (3)*
	Acute abdomen, except gastrointestinal bleeding, *n* (%)	224 (3)	25 (4)*
	Gastrointestinal bleeding, *n* (%)	280 (3)	16 (3)
	Liver failure, *n* (%)	292 (4)	5 (1)**
	Pancreatitis/cholangitis, *n* (%)	79 (1)	10 (2)
	Psychiatric disease, *n* (%)	11 (0)	0 (0)
	Haemorrhage, other, *n* (%)	45 (1)	0 (0)
	Trauma, except traumatic brain injury, *n* (%)	602 (7)	73 (13)***
	Traumatic brain injury, *n* (%)	262 (3)	12 (2)
	Surgical and medical complications, *n* (%)	152 (2)	7 (1)
	Transplantation, *n* (%)	351 (4)	0 (0)***
	Other, *n* (%)	33 (0)	0 (0)
	Postoperative care, not specified, *n* (%)	389 (5)	5 (1)***

Data are presented as mean ± standard deviation or median (interquartile range)

*SAPS* Simplified Acute Physiologic Score, *ICU* intensive care unit, *NEMS* Nine Equivalents of Nursing Manpower Use Score, *SAH* subarachnoid haemorrhage, *COPD* chronic obstructive pulmonary disease. * *p*<0.05, ** *p*<0.01, *** *p*<0.001

### Primary analysis

The crude 90-day mortality was higher among patients transferred due to the need for ICU beds (33.0%; 189/573) than among non-transferred patients (27.0%; 2188/8106, *p* = 0.002). In the stepwise multivariable logistic regression analysis, independent variables associated with an increased risk of death at 90 days were a higher age, SAPS 3 score and mean NEMS as well as ICU diagnoses of cardiac arrest and asphyxia, cerebrovascular event, subarachnoid haemorrhage, traumatic brain injury, COPD/asthma, cardiac disease, aortic rupture or dissection, acute abdomen, gastrointestinal bleeding and respiratory tract infection. Lower mortality was observed in patients with acute surgery or an ICU diagnosis of intoxication, pancreatitis/cholangitis and transplantation. A detailed description of the multivariable analysis is presented in Additional file 1. However, when adjusting for these factors, the risk of death at 90 days did not differ between patients transferred due to a shortage of ICU beds and non-transferred patients; odds ratio 0.923 (95% CI 0.745–1.143, *p* = 0.461) (Table 2).

**Table 2:** The risk of death at 90 days

	Unadjusted			Adjusted^a^		
	OR	95% CI for OR	*p* value	OR	95% CI for OR	*p* value
Transferred vs non-transferred patients	1.33	1.11–1.56	0.002	0.923	0.75–1.14	0.461

^a^Comparison of the risk of death at 90 days after ICU admission between patients transferred due to a shortage of ICU beds and non-transferred patients. There was no difference after adjustment for age, SAPS 3 score, mean NEMS, acute surgery, and ICU diagnosis of cardiac arrest and asphyxia, cerebrovascular event except for SAH, intoxication, transplantation, traumatic brain injury, COPD/asthma, cardiac disease except for cardiac arrest, SAH, pancreatitis/cholangitis, aortic rupture or dissection, gastrointestinal bleeding, acute abdomen except gastrointestinal bleeding, and respiratory tract infection

*OR* odds ratio, *CI* confidence interval, *ICU* intensive care unit

### Secondary analyses

**Fig. 2:**
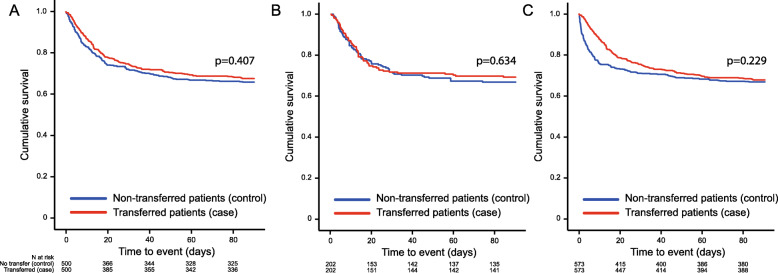
Mortality in transferred patients compared to (**a**) patients matched according to primary ICU diagnosis based on the first three letters of the ICD code, SAPS 3 score, age and time on ward; (**b**) patients matched according to SOFA score, ICU diagnosis in categories, and age; and (**c**) patients matched according to propensity score calculated on the chance of being transferred. *ICU* intensive care unit, *N* number

In the first sensitivity analysis, transferred and non-transferred patients were matched according to the first three letters in the ICD code for the primary ICU diagnosis, age, SAPS 3 score and ICU days, as described in the methods. Of the 573 patients transferred due to the need for ICU beds, 500 (87%) were matched. There was no difference in mortality over time between the transferred patients and the non-transferred matched controls (*p* = 0.407, Fig. 2a).

In the second sensitivity analysis, transferred and non-transferred patients were matched according to SOFA score on day of discharge, ICU diagnosis and age as described in the methods. Of the 238 patients with an available SOFA score who were transferred due to the need for ICU beds, 202 were matched (85%). There was no difference in mortality over time between the transferred patients and the non-transferred matched controls (*p* = 0.634, Fig. 2b).

In the third sensitivity analysis, patients were matched according to propensity score. The propensity score was calculated on the chance of being transferred, as described in the methods. All patients transferred due to the need for ICU beds were matched. There was no difference in mortality over time between the transferred patients and the non-transferred matched controls (*p* = 0.227, Fig. 2c).

There was no difference as to variables on which the matching was based on between transferred and matched non-transferred patients. The baseline characteristics of both these groups are presented in the Additional data.

## Discussion

The main finding of this study was that critically ill patients who were transferred to another ICU due to a shortage of ICU beds in a tertiary centre did not exhibit an increased risk of death compared to that of matched non-transferred patients when adjusting for baseline variables.

The scope of this study was to compare patients transferred due to a shortage of beds in the referring unit to non-transferred patients with a similar degree of vital organ dysfunction. Although there are reports of a higher mortality after ICU transfer due to a shortage of beds compared to transfers for other reasons [10], to the best of our knowledge, there is no analysis similar to ours in the literature. While there are some reports of inter-hospital transfer of patients *to* a tertiary centre [11, 14, 15], the effects of inter-hospital transfer *from* a tertiary centre have not been studied.

In the present era of increased centralization of specialized care, the number of outbound inter-hospital transfers from tertiary centres is high, and their potential effect on mortality is an important issue. Apart from the interest in avoiding excess mortality that comes with refusal of ICU admittance [16], availability is expected for the next critically ill patient in need of specialized care. Efficient use of resources in such a tertiary ICU does not allow final mobilization or boarding of patients who can be cared for in other ICUs [17]. Indeed, patients not in need of tertiary care are not necessarily kept for longer time in our CICU.

One speculative reason for the lack of effect of transfer due to a shortage of ICU beds in our unit on mortality may be that the patient selection is careful and aimed at not transporting unstable patients with a poor prognosis. Notwithstanding, there are many potential negative effects on the patient from inter-hospital transfer. The transport itself might be hazardous with a risk of complications [6, 7]. Medical treatment is halted, and the patients might need intubation for safe transportation [4, 5]. Assessment by a new team might change the attitude of the patient and include restrictions in care [8, 9]. Despite all these potentially negative factors, such transfers did not lead to increased patient mortality in the present study.

There may also be positive effects of ICU-to-ICU transfer. A high total ICU workload has been identified as a factor associated with an increased risk of death [18, 19]. Thus, a transfer from a busier to a quieter ICU could be beneficial. Furthermore, it appears safer to transfer a patient to another ICU than to submit him or her to a premature discharge to the ward [20], which might be the alternative when there is a shortage of ICU resources.

### Strengths and limitations

In this study, we aimed to compare transferred patients to similar patients who remained in the ICU for further care. Since multivariable adjustments and patient matching are a potential source of bias and error, we analysed the material in four different ways and reported all the analyses. In the primary analysis, the risk of death was adjusted for factors that were identified to affect mortality. We included demographic variables, risk score data and patient workload. We used the primary ICU diagnosis in the analysis since different diagnoses have different prognoses. The main disadvantage with this approach was that the ICU diagnoses were categorised, and there could be a significant difference in mortality within each group. For this reason, we performed an analysis in which we matched patients according to the first three characters in the ICD code. As there is a chance that the status of the patient at the time of transfer is important, we also performed an analysis in which we matched patients according to the SOFA score on the day of transfer to patients with similar SOFA scores who remained on the ward. Since there were missing matches in the two latter analyses, we also performed a propensity-score-matched analysis in which patients were matched on the risk of being transferred. In spite of the shortcomings of our data, the fact that all these sensitivity analyses showed similar results supports the main finding that ICU transfer due to a shortage of ICU beds was not associated with an increased risk of short-term death. The major limitation of the present study is that it represents a single centre cohort, and generalizability is limited. Even though the quality of intensive care was very even and according to national standards in the receiving hospitals, the interpretation of the result should be limited to an indicating that patients *can be* safely transferred from a tertiary centre and cared for in another ICU. Transferable patients should be carefully selected in that the receiving hospital has the competence and resources necessary to continue the planned care. Transports need to be carried out in a safe manner with the right competence and equipment. The validity of our results is limited to a 90-day mortality. Other effects, such as total ICU stay, complications and post-ICU quality of life, could not be evaluated in this study.

## Conclusion

In conclusion, the present study suggests that patient mortality is not increased by transfer induced by a shortage of ICU beds in a tertiary centre. Patients selected for transfer should be stable enough to withstand transport and not be expected to have a poor prognosis. Respecting these principles, transfer between ICUs during ongoing intensive care can be justified, albeit not desirable.

## Supplementary Information

**Additional file 1.** Detailed description of statistical analyses and data. Detailed description of multivariable analysis of demographic variables and ICU-diagnoses associated with 90 days mortality. Detailed description of multivariable model building. Tables over transferred cases and matched controls. Complete list of ICD codes and the corresponding ICU diagnosis.

## Data Availability

The datasets used and/or analysed during the current study are available from the corresponding author on reasonable request.
