# Supplementary material for: The mortality of critically ill patients was not associated with inter-hospital transfer due to a shortage of ICU beds - a single-centre retrospective analysis
Source: J Intensive Care. 2020 Oct 30;8:82. doi: 10.1186/s40560-020-00501-z (PMC7598233; doi:10.1186/s40560-020-00501-z)
Supplement: Supplementary file 1 — Additional file 1. Detailed description of statistical analyses and data. Detailed description of multivariable analysis of demographic variables and ICU-diagnoses associated with 90 days mortality. Detailed description of multivariable model building. Tables over transferred cases and matched controls. Complete list of ICD codes and the corresponding ICU diagnosis. [file 40560_2020_501_MOESM1_ESM.pdf]

Final logistic regression model for death at 90 days

Variable	OR	95% CI for OR	p-value
SAPS 3	1.08	1.07 - 1.08	<0.001
Age	1.02	1.01 - 1.02	<0.001
Acute surgery	0.80	0.70 - 0.92	0.001
Mean NEMS-score	1.00	1.00 - 1.01	0.004
Cardiac arrest and asfyxia	3.56	2.92 - 4.33	<0.001
Cerebrovascular event, except SAH	3.58	2.65 - 4.85	<0.001
Intoxication	0.41	0.25 - 0.65	<0.001
Transplantation	0.25	0.14 - 0.44	<0.001
Traumatic brain injury	2.11	1.55 - 2.86	<0.001
COPD/asthma	1.61	1.34 - 1.94	<0.001
Cardiac disease, except cardiac arrest	1.68	1.32 - 2.14	<0.001
Subarachnoid hemorrhage	2.51	1.39 - 4.55	0.002
Pancreatitis/cholangitis	0.48	0.27 - 0.86	0.013
Acute aortic rupture or dissection	1.76	1.23 - 2.52	0.002
Gastrointestinal bleeding	1.50	1.12 - 2.00	0.007
Acute abdomen, except gastrointestinal bleeding	1.52	1.12 - 2.07	0.007
Respiratory tract infection	1.36	1.06 - 1.75	0.015

OR; odds-ratio, CI; confidence intervall

Stepwise multivariable modelling

	Block 1					Block 2													
	Step 1	Step 2	Step 3	Step 4	Step 5	Step 6	Step 7	Step 8	Step 9	Step 10	Step 11	Step 12	Step 13	Stepl 14	Step 15	Step 16	Step 17	Step 18	Step 19
SAPS 3	<0.001	<0.001	<0.001	<0.001	<0.001	<0.001	<0.001	<0.001	<0.001	<0.001	<0.001	<0.001	<0.001	<0.001	<0.001	<0.001	<0.001	<0.001	<0.001
Age	<0.001	<0.001	<0.001	<0.001	<0.001	<0.001	<0.001	<0.001	<0.001	<0.001	<0.001	<0.001	<0.001	<0.001	<0.001	<0.001	<0.001	<0.001	<0.001
Acute surgery	<0.001	<0.001	<0.001	<0.001	<0.001	<0.001	<0.001	<0.001	<0.001	<0.001	0.002	0.004	0.004	0.005	0.005	0.003	0.002	0.001	0.001
Mean NEMS-score	<0.001	0.018	0.006	0.040	0.040	0.040	0.009	0.003	0.011	0.006	0.004	0.004	0.004	0.003	0.002	0.003	0.002	0.003	0.004
Elective surgey	<0.001	0.117	0.511	0.544	0.620														
Sex	0.391	0.760	0.383	0.318	0.312														
Cardiac arrest and asfyxia						<0.001	<0.001	<0.001	<0.001	<0.001	<0.001	<0.001	<0.001	<0.001	<0.001	<0.001	<0.001	<0.001	<0.001
Cerebrovascular event, except SAH						<0.001	<0.001	<0.001	<0.001	<0.001	<0.001	<0.001	<0.001	<0.001	<0.001	<0.001	<0.001	<0.001	<0.001
Intoxication						<0.001	<0.001	<0.001	<0.001	<0.001	<0.001	<0.001	<0.001	<0.001	<0.001	<0.001	<0.001	<0.001	<0.001
Transplantation						<0.001	<0.001	<0.001	<0.001	<0.001	<0.001	<0.001	<0.001	<0.001	<0.001	<0.001	<0.001	<0.001	<0.001
Traumatic brain injury						0.003	<0.001	<0.001	<0.001	<0.001	<0.001	<0.001	<0.001	<0.001	<0.001	<0.001	<0.001	<0.001	<0.001
COPD/asthma						0.047	<0.001	<0.001	<0.001	0.001	<0.001	<0.001	<0.001	<0.001	<0.001	<0.001	<0.001	<0.001	<0.001
Cardiac disease, except cardiac arrest						0.091	0.008	0.002	0.003	0.007	0.004	0.001	0.001	<0.001	0.001	<0.001	<0.001	<0.001	<0.001
Subarachnoid hemorrhage						0.033	0.014	0.009	0.013	0.017	0.014	0.008	0.004	0.004	0.004	0.004	0.004	0.004	0.002
Pancreatitis/cholangitis						<0.001	0.001	0.002	0.002	0.001	0.001	0.003	0.005	0.005	0.005	0.005	0.007	0.009	0.013
Acute aortic rupture or dissection						0.105	0.043	0.022	0.024	0.048	0.037	0.018	0.009	0.007	0.010	0.010	0.006	0.004	0.002
Gastrointestinal bleeding						0.52	0.155	0.075	0.105	0.187	0.137	0.063	0.031	0.026	0.034	0.022	0.022	0.013	0.007
Acute abdomen, except gastrointestinal bleeding						0.43	0.145	0.075	0.087	0.174	0.134	0.065	0.033	0.028	0.037	0.023	0.014	0.014	0.007
Respiratory tract infection						0.949	0.354	0.192	0.285	0.407	0.299	0.129	0.063	0.049	0.07	0.048	0.028	0.015	0.015
Neurological disease						<0.001	0.002	0.007	0.003	0.002	0.003	0.009	0.019	0.024	0.018	0.024	0.036	0.054	0.096
Other						0.725	0.536	0.471	0.509	0.54	0.503	0.42	0.366	0.351	0.374	0.349	0.321	0.291	0.248
Hematological disease						0.781	0.521	0.45	0.521	0.569	0.52	0.437	0.387	0.37	0.393	0.376	0.346	0.319	0.275
Infection/sepsis, except respiratory tract infection						0.001	0.08	0.235	0.141	0.064	0.121	0.391	0.731	0.841	0.647	0.824	0.923	0.644	0.304
Liver failure						0.004	0.071	0.136	0.08	0.03	0.047	0.099	0.155	0.179	0.138	0.165	0.22	0.294	0.428
Postoperative care, not specified						0.565	0.837	0.935	0.921	0.709	0.812	0.968	0.79	0.731	0.785	0.662	0.678	0.565	0.449
Renal/urologic disease						0.37	0.763	0.932	0.841	0.737	0.823	0.977	0.831	0.791	0.856	0.789	0.703	0.612	0.491
Arterial disease, not ruptured aorta						0.071	0.113	0.174	0.158	0.096	0.114	0.173	0.242	0.263	0.238	0.305	0.373	0.472	0.581
Circulatory shock, other						0.06	0.168	0.24	0.194	0.146	0.177	0.257	0.331	0.356	0.321	0.363	0.422	0.492	0.597
Endocrinal disease						0.019	0.08	0.141	0.09	0.07	0.097	0.178	0.264	0.3	0.262	0.314	0.389	0.476	0.628
Psyciatric disease						0.769	0.728	0.699	0.746	0.77	0.75	0.72	0.7	0.689	0.694	0.682	0.667	0.653	0.63
Hemorrhage, not specified						0.887	0.937	0.854	0.929	0.993	0.96	0.875	0.814	0.791	0.814	0.78	0.737	0.693	0.634
Respiratory tract disease, except infection or COPD/asthma						0.475	0.687	0.826	0.758	0.664	0.727	0.881	0.994	0.957	0.998	0.924	0.843	0.752	0.643
Trauma, except traumatic brain injury						0.184	0.458	0.652	0.433	0.264	0.351	0.544	0.704	0.778	0.706	0.811	0.941	0.917	0.716
Malignancy						0.179	0.345	0.473	0.374	0.308	0.368	0.523	0.657	0.706	0.654	0.738	0.835	0.943	0.904
Musculoskeletal disease						0.999	0.999	0.999	0.999	0.999	0.999	0.999	0.999	0.999	0.999	0.999	0.999	0.999	0.999

The model was built with a step-wise method in two blocks. In the first block, SAPS 3, age, sex, surgical status and NEMS score were included. In the second step, the primary ICD diagnoses were tested. Variables not in the model were tested in the model one at the time. The variable with the lowest p-value at inclusion in the model were included at each step. This was repeated until there were no remaining variables with a p-value <0.05. Yellow fields represents variables in the model at each step. White fields represent variables tested one at the time in the model.

Case-control matching according to first three letters of ICD-code of primary ICU-diagnosis, age, SAPS 3 score and ICU-stay

Category	Variable	Matched cohort (n=500)	Transferred patients (n=500)
Demographics	Female sex, n (%)	178 (36)	187 (37)
	Age, years	67 (52 - 74)	67 (52 - 74)
ICU data	SAPS 3 score	61 ± 14	62 ± 14
	ICU days, days	5 (3 - 10)	3 (1 - 5)***
	Mean NEMS	34 (29 - 39)	38 (34 - 43)***
Surgery	No surgery, n (%)	335 (67)	346 (69)
	Acute surgery, n (%)	113 (23)	122 (24)
	Elective surgery, n (%)	52 (10)	32 (6)*
Primary ICU diagnosis in categories	Infection/sepsis, except respiratory tract infection, n (%)	79 (16)	83 (17)
	Malignancy, n (%)	0 (0)	0 (0)
	Haematological disease, n (%)	0 (0)	0 (0)
	Endocrinal disease, n (%)	14 (3)	14 (3)
	Intoxication, n (%)	14 (3)	14 (3)
	Neurological disease, n (%)	13 (3)	13 (3)
	Cardiac disease, except cardiac arrest, n (%)	18 (4)	18 (4)
	Cardiac arrest and asphyxia, n (%)	69 (14)	69 (14)
	Subarachnoid haemorrhage, n (%)	0 (0)	0 (0)
	Cerebrovascular event, except SAH, n (%)	6 (1)	6 (1)
	Acute aortic rupture or dissection, n (%)	4 (1)	4 (1)
	Arterial disease, not ruptured aorta, n (%)	2 (0)	2 (0)
	Musculoskeletal disease, n (%)	0 (0)	0 (0)
	Circulatory shock, other, n (%)	9 (2)	3 (1)
	Respiratory tract infection, n (%)	54 (11)	54 (11)
	COPD/asthma, n (%)	66 (13)	66 (13)
	Respiratory tract disease, except infection or COPD/asthma, n (%)	3 (1)	3 (1)
	Renal/urologic disease, n (%)	11 (2)	11 (2)
	Acute abdomen, except gastrointestinal bleeding, n (%)	22 (4)	22 (4)
	Gastrointestinal bleeding, n (%)	15 (3)	15 (3)
	Liver failure, n (%)	4 (1)	4 (1)
	Pancreatitis/cholangitis, n (%)	9 (2)	9 (2)
	Psychiatric disease, n (%)	0 (0)	0 (0)
	Haemorrhage, other, n (%)	0 (0)	0 (0)
	Trauma, except traumatic brain injury, n (%)	67 (13)	67 (13)
	Traumatic brain injury, n (%)	4 (1)	6 (1)
	Surgical and medical complications, n (%)	0 (0)	0 (0)
	Transplantation, n (%)	12 (2)	12 (2)
	Other, n (%)	0 (0)	0 (0)
	Postoperative care, not specified, n (%)	5 (1)	5 (1)

* p<0.05, ** p<0.01, *** p<0.001

Case-control matching according to SOFA-score, age and ICU-diagnosis in categories

Category	Variable	Matched controls (n=202)	Transferred patients (n=202)
Demographics	Female sex, n (%)	74 (37)	82 (41)
	Age, years	66 (52 - 76)	65 (49 - 74)
ICU data	SAPS 3 score	63 ± 15	63 ± 14
	ICU days, days	4 (3 - 9)	3 (2 - 4)***
	Mean NEMS	37 (33 - 40)	44 (37 - 58)***
Surgery	No surgery, n (%)	133 (66)	161 (80)**
	Acute surgery, n (%)	61 (30)	38 (19)**
	Elective surgery, n (%)	8 (4)	3 (1)
Primary ICU diagnosis in categories	Infection/sepsis, except respiratory tract infection, n (%)	45 (22)	45 (22)
	Malignancy, n (%)	0 (0)	0 (0)
	Haematological disease, n (%)	0 (0)	0 (0)
	Endocrinal disease, n (%)	5 (2)	5 (2)
	Intoxication, n (%)	6 (3)	6 (3)
	Neurological disease, n (%)	11 (5)	11 (5)
	Cardiac disease, except cardiac arrest, n (%)	5 (2)	5 (2)
	Cardiac arrest and asphyxia, n (%)	27 (13)	27 (13)
	Subarachnoid haemorrhage, n (%)	0 (0)	0 (0)
	Cerebrovascular event, except SAH, n (%)	2 (1)	2 (1)
	Acute aortic rupture or dissection, n (%)	0 (0)	0 (0)
	Arterial disease, not ruptured aorta, n (%)	0 (0)	0 (0)
	Musculoskeletal disease, n (%)	0 (0)	0 (0)
	Circulatory shock, other, n (%)	1 (0)	1 (0)
	Respiratory tract infection, n (%)	28 (14)	28 (14)
	COPD/asthma, n (%)	23 (11)	23 (11)
	Respiratory tract disease, except infection or COPD/asthma, n (%)	1 (0)	1 (0)
	Renal/urologic disease, n (%)	6 (3)	6 (3)
	Acute abdomen, except gastrointestinal bleeding, n (%)	7 (3)	7 (3)
	Gastrointestinal bleeding, n (%)	3 (1)	3 (1)
	Liver failure, n (%)	1 (0)	1 (0)
	Pancreatitis/cholangitis, n (%)	2 (1)	2 (1)
	Psychiatric disease, n (%)	0 (0)	0 (0)
	Haemorrhage, other, n (%)	0 (0)	0 (0)
	Trauma, except traumatic brain injury, n (%)	20 (10)	20 (10)
	Traumatic brain injury, n (%)	5 (2)	5 (2)
	Surgical and medical complications, n (%)	3 (1)	3 (1)
	Transplantation, n (%)	0 (0)	0 (0)
	Other, n (%)	0 (0)	0 (0)
	Postoperative care, not specified, n (%)	1 (0)	1 (0)

* p<0.05, ** p<0.01, *** p<0.001

Propensity score matched case/controls

Category	Variable	Matched cohort (n=573)	Transferred patients (n=573)
Demographics	Female sex, n (%)	231 (40)	224 (39)
	Age, years	66 (52 - 74)	66 (50 - 74)
ICU data	SAPS 3 score	63 ± 16	62 ± 15
	ICU days, days	2 (1 - 5)	3 (2 - 5)
	Mean NEMS	36 (31 - 44)	38 (34 - 43)**
Surgery	No surgery, n (%)	353 (62)	399 (70)
	Acute surgery, n (%)	158 (28)	138 (24)
	Elective surgery, n (%)	62 (11)	36 (6)**
Primary ICU diagnosis	Infection/sepsis, except respiratory tract infection, n (%)	64 (11)	97 (17)**
	Malignancy, n (%)	21 (4)	0 (0)***
	Haematological disease, n (%)	3 (1)	0 (0)
	Endocrinal disease, n (%)	21 (4)	16 (3)
	Intoxication, n (%)	14 (2)	24 (4)
	Neurological disease, n (%)	22 (4)	20 (3)
	Cardiac disease, except cardiac arrest, n (%)	37 (6)	19 (3)*
	Cardiac arrest and asphyxia, n (%)	72 (13)	70 (12)
	Subarachnoid haemorrhage, n (%)	4 (1)	0 (0)
	Cerebrovascular event, except SAH, n (%)	20 (3)	7 *
	Acute aortic rupture or dissection, n (%)	9 (2)	5 (1)
	Arterial disease, not ruptured aorta, n (%)	11 (2)	2 (0)*
	Musculoskeletal disease, n (%)	0 (0)	0 (0)
	Circulatory shock, other, n (%)	6 (1)	3 (1)
	Respiratory tract infection, n (%)	24 (4)	69 (12)***
	COPD/asthma, n (%)	54 (9)	67 (12)
	Respiratory tract disease, except infection or COPD/asthma, n (%)	7 (1)	6 (1)
	Renal/urologic disease, n (%)	6 (1)	15 (3)
	Acute abdomen, except gastrointestinal bleeding, n (%)	16 (3)	25 (4)
	Gastrointestinal bleeding, n (%)	18 (3)	16 (3)
	Liver failure, n (%)	29 (5)	5 (1)***
	Pancreatitis/cholangitis, n (%)	10 (2)	10 (2)
	Psychiatric disease, n (%)	0 (0)	0 (0)
	Haemorrhage, other, n (%)	2 (0)	0 (0)
	Trauma, except traumatic brain injury, n (%)	37 (6)	73 (13)***
	Traumatic brain injury, n (%)	24 (4)	12 (2)
	Surgical and medical complications, n (%)	16 (3)	7 (1)
	Transplantation, n (%)	19 (3)	0 (0)***
	Other, n (%)	4 (1)	0 (0)
	Postoperative care, not specified, n (%)	3 (1)	5 (1)

* p<0.05, ** p<0.01, *** p<0.001

Primary ICD-codes and the corresponding ICU-diagnosis

ICD-code	ICU-diagnosis	Number of patients		ICD-text
		No transfer	Transferred due to shortage of ICU beds	
K209	Acute abdomen, except gastrointestinal bleeding	1	0	Esophagitis, unspecified
K223	Acute abdomen, except gastrointestinal bleeding	18	1	Perforation of esophagus
K238	Acute abdomen, except gastrointestinal bleeding	1	0	Disorders of esophagus in diseases classified elsewhere
K250	Acute abdomen, except gastrointestinal bleeding	10	2	Acute gastric ulcer with hemorrhage
K251	Acute abdomen, except gastrointestinal bleeding	18	2	Acute gastric ulcer with perforation
K260	Acute abdomen, except gastrointestinal bleeding	0	0	Acute duodenal ulcer with hemorrhage
K261	Acute abdomen, except gastrointestinal bleeding	1	0	Acute duodenal ulcer with perforation
K270	Acute abdomen, except gastrointestinal bleeding	1	0	Acute peptic ulcer, site unspecified, with hemorrhage
K271	Acute abdomen, except gastrointestinal bleeding	0	0	Acute peptic ulcer, site unspecified, with perforation
K279	Acute abdomen, except gastrointestinal bleeding	17	2	Peptic ulc, site unsp, unsp as ac or chr, w/o hemor or perf
K352	Acute abdomen, except gastrointestinal bleeding	1	0	Acute appendicitis with generalized peritonitis
K403	Acute abdomen, except gastrointestinal bleeding	1	0	Unilateral inguinal hernia, with obstruction, w/o gangrene
K430	Acute abdomen, except gastrointestinal bleeding	1	0	Incisional hernia with obstruction, without gangrene
K460	Acute abdomen, except gastrointestinal bleeding	2	0	Unsp abdominal hernia with obstruction, without gangrene
K461	Acute abdomen, except gastrointestinal bleeding	1	0	Unspecified abdominal hernia with gangrene
K519	Acute abdomen, except gastrointestinal bleeding	1	0	Ulcerative colitis, unspecified
K529	Acute abdomen, except gastrointestinal bleeding	3	0	Noninfective gastroenteritis and colitis, unspecified
K550	Acute abdomen, except gastrointestinal bleeding	30	2	Acute vascular disorders of intestine
K563	Acute abdomen, except gastrointestinal bleeding	1	0	Gallstone ileus
K565	Acute abdomen, except gastrointestinal bleeding	2	0	Intestinal adhesions w obst (postprocedural) (postinfection)
K567	Acute abdomen, except gastrointestinal bleeding	46	6	Ileus, unspecified
K570	Acute abdomen, except gastrointestinal bleeding	1	0	Diverticulitis of small intestine w perforation and abscess
K590	Acute abdomen, except gastrointestinal bleeding	1	0	Constipation
K630	Acute abdomen, except gastrointestinal bleeding	1	0	Abscess of intestine
K631	Acute abdomen, except gastrointestinal bleeding	37	8	Perforation of intestine (nontraumatic)
K650	Acute abdomen, except gastrointestinal bleeding	21	2	Generalized (acute) peritonitis
K919	Acute abdomen, except gastrointestinal bleeding	1	0	Oth postprocedural complications and disorders of dgstv sys
K928	Acute abdomen, except gastrointestinal bleeding	1	0	Other specified diseases of the digestive system
R104	Acute abdomen, except gastrointestinal bleeding	4	0	Periumbilical pain
R198	Acute abdomen, except gastrointestinal bleeding	1	0	Oth symptoms and signs involving the dgstv sys and abdomen
I710	Acute aortic rupture or dissection	70	0	Dissection of aorta
I711	Acute aortic rupture or dissection	1	0	Thoracic aortic aneurysm, ruptured
I713	Acute aortic rupture or dissection	10	0	Abdominal aortic aneurysm, ruptured

I718	Acute aortic rupture or dissection	101	5	Aortic aneurysm of unspecified site, ruptured
I702	Arterial disease, not ruptured aorta	1	0	Atherosclerosis of native arteries of the extremities
I708	Arterial disease, not ruptured aorta	2	0	Atherosclerosis of other arteries
I709	Arterial disease, not ruptured aorta	2	0	Other and unspecified atherosclerosis
I712	Arterial disease, not ruptured aorta	1	0	Thoracic aortic aneurysm, without rupture
I714	Arterial disease, not ruptured aorta	5	0	Abdominal aortic aneurysm, without rupture
I716	Arterial disease, not ruptured aorta	0	0	Thoracoabdominal aortic aneurysm, without rupture
I719	Arterial disease, not ruptured aorta	45	1	Aortic aneurysm of unspecified site, without rupture
I724	Arterial disease, not ruptured aorta	1	0	Aneurysm of artery of lower extremity
I729	Arterial disease, not ruptured aorta	1	0	Aneurysm of unspecified site
I739	Arterial disease, not ruptured aorta	36	0	Peripheral vascular disease, unspecified
I740	Arterial disease, not ruptured aorta	2	0	Embolism and thrombosis of abdominal aorta
I742	Arterial disease, not ruptured aorta	2	0	Embolism and thrombosis of arteries of the upper extremities
I743	Arterial disease, not ruptured aorta	11	1	Embolism and thrombosis of arteries of the lower extremities
I745	Arterial disease, not ruptured aorta	1	0	Embolism and thrombosis of iliac artery
I748	Arterial disease, not ruptured aorta	1	0	Embolism and thrombosis of other arteries
I749	Arterial disease, not ruptured aorta	131	0	Embolism and thrombosis of unspecified artery
I779	Arterial disease, not ruptured aorta	0	0	Disorder of arteries and arterioles, unspecified
I798	Arterial disease, not ruptured aorta	1	0	Oth disord of art,arterioles & capilare in dis classd elswhr
I829	Arterial disease, not ruptured aorta	8	0	Chronic embolism and thrombosis of deep vn unsp up extrem
I469	Cardiac arrest and asfyxia	635	70	Cardiac arrest, cause unspecified
P284	Cardiac arrest and asfyxia	1	0	Other apnea of newborn
R090	Cardiac arrest and asfyxia	3	0	Asphyxia and hypoxemia
R092	Cardiac arrest and asfyxia	4	0	Respiratory arrest
T719	Cardiac arrest and asfyxia	4	0	Asphyxiation due to unspecified cause
T751	Cardiac arrest and asfyxia	8	0	Unspecified effects of drowning and nonfatal submersion
G931	Cardiac disease, except cardiac arrest	7	0	Anoxic brain damage, not elsewhere classified
I109	Cardiac disease, except cardiac arrest	8	0	Essential (primary) hypertension
I110	Cardiac disease, except cardiac arrest	1	0	Hypertensive heart disease with heart failure
I152	Cardiac disease, except cardiac arrest	1	0	Hypertension secondary to endocrine disorders
I212	Cardiac disease, except cardiac arrest	1	0	ST elevation (STEMI) myocardial infarction of other sites
I214	Cardiac disease, except cardiac arrest	0	0	Non-ST elevation (NSTEMI) myocardial infarction
I219	Cardiac disease, except cardiac arrest	45	7	Non-ST elevation (NSTEMI) myocardial infarction
I238	Cardiac disease, except cardiac arrest	1	0	Oth current complications following AMI
I241	Cardiac disease, except cardiac arrest	4	1	Dressler's syndrome
I249	Cardiac disease, except cardiac arrest	2	0	Acute ischemic heart disease, unspecified
I259	Cardiac disease, except cardiac arrest	1	0	Chronic ischemic heart disease, unspecified
I260	Cardiac disease, except cardiac arrest	8	0	Pulmonary embolism with acute cor pulmonale
I269	Cardiac disease, except cardiac arrest	82	2	Pulmonary embolism without acute cor pulmonale
I270	Cardiac disease, except cardiac arrest	2	0	Primary pulmonary hypertension

I309	Cardiac disease, except cardiac arrest	1	0	Acute pericarditis, unspecified
I319	Cardiac disease, except cardiac arrest	6	0	Disease of pericardium, unspecified
I330	Cardiac disease, except cardiac arrest	1	0	Acute and subacute infective endocarditis
I340	Cardiac disease, except cardiac arrest	3	0	Nonrheumatic mitral (valve) insufficiency
I350	Cardiac disease, except cardiac arrest	5	0	Nonrheumatic aortic (valve) stenosis
I389	Cardiac disease, except cardiac arrest	0	0	Endocarditis, valve unspecified
I421	Cardiac disease, except cardiac arrest	1	0	Obstructive hypertrophic cardiomyopathy
I429	Cardiac disease, except cardiac arrest	3	0	Cardiomyopathy, unspecified
I442	Cardiac disease, except cardiac arrest	5	0	Atrioventricular block, complete
I455	Cardiac disease, except cardiac arrest	1	0	Other specified heart block
I459	Cardiac disease, except cardiac arrest	2	0	Conduction disorder, unspecified
I489	Cardiac disease, except cardiac arrest	20	0	Unspecified atrial fibrillation and atrial flutter
I490	Cardiac disease, except cardiac arrest	2	0	Ventricular fibrillation and flutter
I495	Cardiac disease, except cardiac arrest	1	0	Sick sinus syndrome
I499	Cardiac disease, except cardiac arrest	6	0	Cardiac arrhythmia, unspecified
I500	Cardiac disease, except cardiac arrest	4	0	Heart failure
I501	Cardiac disease, except cardiac arrest	59	5	Left ventricular failure
I509	Cardiac disease, except cardiac arrest	65	4	Heart failure, unspecified
I519	Cardiac disease, except cardiac arrest	3	0	Heart disease, unspecified
R001	Cardiac disease, except cardiac arrest	4	0	Bradycardia, unspecified
R570	Cardiac disease, except cardiac arrest	33	0	Cardiogenic shock
Z034	Cardiac disease, except cardiac arrest	2	0	Encntr for medical obs for susp diseases and cond ruled out
I610	Cerebrovascular event, except SAH	1	1	Nontraumatic intcrbl hemorrhage in hemisphere, subcortical
I611	Cerebrovascular event, except SAH	1	0	Nontraumatic intcrbl hemorrhage in hemisphere, cortical
I612	Cerebrovascular event, except SAH	45	2	Nontraumatic intracerebral hemorrhage in hemisphere, unsp
I613	Cerebrovascular event, except SAH	9	0	Nontraumatic intracerebral hemorrhage in brain stem
I614	Cerebrovascular event, except SAH	5	1	Nontraumatic intracerebral hemorrhage in cerebellum
I619	Cerebrovascular event, except SAH	7	0	Nontraumatic intracerebral hemorrhage, unspecified
I620	Cerebrovascular event, except SAH	14	1	Nontraumatic subdural hemorrhage
I629	Cerebrovascular event, except SAH	40	0	Nontraumatic intracranial hemorrhage, unspecified
I630	Cerebrovascular event, except SAH	2	0	Cerebral infarction due to thrombosis of precerb arteries
I633	Cerebrovascular event, except SAH	4	0	Cerebral infarction due to thrombosis of cerebral arteries
I634	Cerebrovascular event, except SAH	1	0	Cerebral infarction due to embolism of cerebral arteries
I635	Cerebrovascular event, except SAH	1	0	Cerebral infrc due to unsp occls or stenosis of cerebral art
I636	Cerebrovascular event, except SAH	11	0	Cerebral infrc due to cerebral venous thombos, nonpyogenic
I638	Cerebrovascular event, except SAH	4	0	Other cerebral infarction
I639	Cerebrovascular event, except SAH	69	1	Cerebral infarction, unspecified
I652	Cerebrovascular event, except SAH	4	0	Occlusion and stenosis of carotid artery
I669	Cerebrovascular event, except SAH	7	0	Occlusion and stenosis of unspecified cerebral artery
I679	Cerebrovascular event, except SAH	6	0	Cerebrovascular disease, unspecified

I691	Cerebrovascular event, except SAH	0	0	Sequelae of nontraumatic intracerebral hemorrhage
I693	Cerebrovascular event, except SAH	0	1	Sequelae of cerebral infarction
I952	Circulatory shock, not specified	1	0	Hypotension due to drugs
I958	Circulatory shock, not specified	4	0	Other hypotension
I959	Circulatory shock, not specified	7	0	Hypotension, unspecified
R571	Circulatory shock, not specified	61	3	Hypovolemic shock
R578	Circulatory shock, not specified	2	0	Other shock
R579	Circulatory shock, not specified	2	0	Shock, unspecified
T782	Circulatory shock, not specified	30	0	Anaphylactic shock, unspecified
T783	Circulatory shock, not specified	1	0	Angioneurotic edema
T784	Circulatory shock, not specified	11	0	Other and unspecified allergy
T811	Circulatory shock, not specified	13	0	Postprocedural shock
J440	COPD/asthma	2	0	Chronic obstructive pulmon disease w acute lower resp infct
J441	COPD/asthma	7	1	Chronic obstructive pulmonary disease w (acute) exacerbation
J448	COPD/asthma	1	0	Chronic obstructive pulmonary disease w (acute) exacerbation
J449	COPD/asthma	73	7	Chronic obstructive pulmonary disease, unspecified
J469	COPD/asthma	14	1	Other asthma
J809	COPD/asthma	36	5	Acute respiratory distress syndrome
J819	COPD/asthma	49	5	Chronic pulmonary edema
J841	COPD/asthma	15	0	Other interstitial pulmonary diseases with fibrosis
J939	COPD/asthma	21	0	Pneumothorax, unspecified
J942	COPD/asthma	1	0	Hemothorax
J948	COPD/asthma	2	0	Other specified pleural conditions
J951	COPD/asthma	4	0	Acute pulmonary insufficiency following thoracic surgery
J952	COPD/asthma	37	2	Acute pulmonary insufficiency following nonthoracic surgery
J960	COPD/asthma	16	1	Acute respiratory failure
J961	COPD/asthma	1	0	Chronic respiratory failure
J969	COPD/asthma	441	45	Respiratory failure, unspecified
J981	COPD/asthma	8	0	Pulmonary collapse
J985	COPD/asthma	2	0	Diseases of mediastinum, not elsewhere classified
J988	COPD/asthma	1	0	Other specified respiratory disorders
J989	COPD/asthma	2	0	Respiratory disorder, unspecified
E035	Endocrinal disease	0	0	Myxedema coma
E040	Endocrinal disease	0	1	Nontoxic diffuse goiter
E055	Endocrinal disease	1	0	Thyrotoxicosis factitia with thyrotoxic crisis or storm
E079	Endocrinal disease	1	0	Disorder of thyroid, unspecified
E100	Endocrinal disease	2	0	Type 1 diabetes mellitus
E101	Endocrinal disease	3	0	Type 1 diabetes mellitus with ketoacidosis
E108	Endocrinal disease	1	0	Type 1 diabetes mellitus with unspecified complications
E111	Endocrinal disease	2	0	Type 2 diabetes mellitus with hyperosmolarity with coma

E111A	Endocrinal disease	1	0	Type 2 diabetes mellitus with hyperosmolarity with coma
E140	Endocrinal disease	15	1	Other specified diabetes mellitus without complications
E141	Endocrinal disease	52	3	Other specified diabetes mellitus without complications
E142	Endocrinal disease	1	0	Other specified diabetes mellitus without complications
E160	Endocrinal disease	1	0	Drug-induced hypoglycemia without coma
E162	Endocrinal disease	1	0	Hypoglycemia, unspecified
E213	Endocrinal disease	1	0	Hyperparathyroidism, unspecified
E222	Endocrinal disease	2	0	Syndrome of inappropriate secretion of antidiuretic hormone
E274	Endocrinal disease	2	0	Other and unspecified adrenocortical insufficiency
E340	Endocrinal disease	2	0	Carcinoid syndrome
E662	Endocrinal disease	1	0	Morbid (severe) obesity with alveolar hypoventilation
E669	Endocrinal disease	1	0	Obesity, unspecified
E869	Endocrinal disease	18	0	Volume depletion, unspecified
E870	Endocrinal disease	8	1	Hyperosmolality and hypernatremia
E871	Endocrinal disease	77	4	Hypo-osmolality and hyponatremia
E871B	Endocrinal disease	1	0	Hypo-osmolality and hyponatremia
E872	Endocrinal disease	22	2	Acidosis
E873	Endocrinal disease	3	0	Alkalosis
E875	Endocrinal disease	25	1	Hyperkalemia
E876	Endocrinal disease	25	1	Hypokalemia
E877	Endocrinal disease	2	0	Fluid overload
E878	Endocrinal disease	17	2	Oth disorders of electrolyte and fluid balance, NEC
R739	Endocrinal disease	1	0	Hyperglycemia, unspecified
K921	Gastrointestinal bleeding	1	0	Melena
K922	Gastrointestinal bleeding	279	16	Gastrointestinal hemorrhage, unspecified
C795	Hematological disease	1	0	Malignant neoplasm of endocrine gland, unspecified
C851	Hematological disease	1	0	Unspecified B-cell lymphoma
C900	Hematological disease	1	0	Multiple myeloma
C920	Hematological disease	1	0	Acute myeloblastic leukemia
C969	Hematological disease	15	0	Unifocal Langerhans-cell histiocytosis
D629	Hematological disease	0	0	Acute posthemorrhagic anemia
D689	Hematological disease	2	0	Coagulation defect, unspecified
D693	Hematological disease	0	0	Immune thrombocytopenic purpura
D695	Hematological disease	1	0	Secondary thrombocytopenia
D709	Hematological disease	1	0	Neutropenia, unspecified
D709C	Hematological disease	1	0	Neutropenia, unspecified
D759	Hematological disease	10	0	Disease of blood and blood-forming organs, unspecified
D849	Hematological disease	1	0	Immunodeficiency, unspecified
N939	Hemorrhage, not specified	4	0	Abnormal uterine and vaginal bleeding, unspecified
O723	Hemorrhage, not specified	2	0	Postpartum coagulation defects

R589	Hemorrhage, not specified	39	0	Hemorrhage, not elsewhere classified
A030	Infection/sepsis, except respiratory tract infection	0	1	Shigellosis due to Shigella dysenteriae
A047	Infection/sepsis, except respiratory tract infection	1	0	Enterocolitis due to Clostridium difficile
A085	Infection/sepsis, except respiratory tract infection	0	1	Viral intestinal infection, unspecified
A099	Infection/sepsis, except respiratory tract infection	2	0	Infectious gastroenteritis and colitis, unspecified
A408	Infection/sepsis, except respiratory tract infection	1	0	Other streptococcal sepsis
A410	Infection/sepsis, except respiratory tract infection	2	0	Sepsis due to Staphylococcus aureus
A415	Infection/sepsis, except respiratory tract infection	2	1	Sepsis due to other Gram-negative organisms
A418	Infection/sepsis, except respiratory tract infection	0	0	Other specified sepsis
A419	Infection/sepsis, except respiratory tract infection	13	2	Sepsis, unspecified organism
A469	Infection/sepsis, except respiratory tract infection	1	0	Erysipelas
A490	Infection/sepsis, except respiratory tract infection	0	0	Staphylococcal infection, unspecified site
A498	Infection/sepsis, except respiratory tract infection	2	0	Other bacterial infections of unspecified site
A499	Infection/sepsis, except respiratory tract infection	22	5	Bacterial infection, unspecified
A869	Infection/sepsis, except respiratory tract infection	1	0	Unspecified viral encephalitis
A879	Infection/sepsis, except respiratory tract infection	1	0	Viral meningitis, unspecified
B004	Infection/sepsis, except respiratory tract infection	3	0	Herpesviral encephalitis
B199	Infection/sepsis, except respiratory tract infection	1	1	Unspecified viral hepatitis without hepatic coma
B349	Infection/sepsis, except respiratory tract infection	1	0	Viral infection, unspecified
B379	Infection/sepsis, except respiratory tract infection	1	0	Candidiasis, unspecified
B499	Infection/sepsis, except respiratory tract infection	1	0	Unspecified mycosis
B549	Infection/sepsis, except respiratory tract infection	1	0	Unspecified malaria
B599	Infection/sepsis, except respiratory tract infection	1	0	Pneumocystosis
B670	Infection/sepsis, except respiratory tract infection	1	0	Echinococcus granulosus infection of liver
B950	Infection/sepsis, except respiratory tract infection	0	1	Streptococcus, group A, causing diseases classd elswhr
B951	Infection/sepsis, except respiratory tract infection	1	0	Streptococcus, group B, causing diseases classd elswhr
B953	Infection/sepsis, except respiratory tract infection	3	0	Streptococcus pneumoniae causing diseases classd elswhr
B955	Infection/sepsis, except respiratory tract infection	1	0	Unsp streptococcus as the cause of diseases classd elswhr
B956	Infection/sepsis, except respiratory tract infection	2	0	Staphylococcus aureus as the cause of diseases classd elswhr
B962	Infection/sepsis, except respiratory tract infection	2	0	Escherichia coli as the cause of diseases classd elswhr
B965	Infection/sepsis, except respiratory tract infection	2	0	Pseudomonas (mallei) causing diseases classd elswhr
B999	Infection/sepsis, except respiratory tract infection	15	1	Unspecified infectious disease
G009	Infection/sepsis, except respiratory tract infection	11	4	Bacterial meningitis, unspecified
G039	Infection/sepsis, except respiratory tract infection	2	0	Meningitis, unspecified
G049	Infection/sepsis, except respiratory tract infection	2	0	Encephalitis, myelitis and encephalomyelitis, unspecified
G060	Infection/sepsis, except respiratory tract infection	1	0	Intracranial abscess and granuloma
G061	Infection/sepsis, except respiratory tract infection	2	0	Intraspinal abscess and granuloma
L033	Infection/sepsis, except respiratory tract infection	1	0	Cellulitis and acute lymphangitis of trunk
L088	Infection/sepsis, except respiratory tract infection	2	0	Oth local infections of the skin and subcutaneous tissue
L089	Infection/sepsis, except respiratory tract infection	5	2	Local infection of the skin and subcutaneous tissue, unsp

L979	Infection/sepsis, except respiratory tract infection	1	0	Non-pressure chronic ulcer of unspecified part of lower leg
M463	Infection/sepsis, except respiratory tract infection	1	0	Infection of intervertebral disc (pyogenic)
M726	Infection/sepsis, except respiratory tract infection	6	7	Necrotizing fasciitis
M869	Infection/sepsis, except respiratory tract infection	0	0	Osteomyelitis, unspecified
N390	Infection/sepsis, except respiratory tract infection	36	4	Urinary tract infection, site not specified
R572	Infection/sepsis, except respiratory tract infection	351	50	Hypovolemic shock
R651	Infection/sepsis, except respiratory tract infection	154	16	SIRS of non-infectious origin
T814	Infection/sepsis, except respiratory tract infection	15	1	Infection following a procedure
F100	Intoxication	51	2	Alcohol related disorders
F104	Intoxication	3	0	Alcohol dependence with unspecified alcohol-induced disorder
F110	Intoxication	5	0	Opioid related disorders
F130	Intoxication	43	2	Sedative, hypnotic, or anxiolytic related disorders
F150	Intoxication	1	0	Other stimulant related disorders
F190	Intoxication	9	1	Other psychoactive substance related disorders
F191	Intoxication	1	0	Other psychoactive substance abuse
F199	Intoxication	1	1	Other psychoactive substance use, unspecified
T391	Intoxication	16	0	4-Aminophenol derivatives
T401	Intoxication	7	0	Poisoning by and adverse effect of heroin
T402	Intoxication	22	0	Poisoning by, adverse effect of and underdosing of opioids
T404	Intoxication	1	0	Synthetic narcotics
T406	Intoxication	33	0	And unsp narcotics
T407	Intoxication	3	1	Cannabis (derivatives)
T409	Intoxication	1	0	And unsp psychodysleptics
T414	Intoxication	5	0	Unsp anesthetic
T423	Intoxication	1	0	Barbiturates
T424	Intoxication	33	0	Benzodiazepines
T426	Intoxication	1	0	Antiepileptic and sedative-hypnotic drugs
T427	Intoxication	32	2	Unsp antiepileptic and sedative-hypnotic drugs
T430	Intoxication	17	1	Tricyclic and tetracyclic antidepressants
T432	Intoxication	8	2	And unsp antidepressants
T434	Intoxication	1	0	Butyrophenone and thiothixene neuroleptics
T435	Intoxication	12	3	And unsp antipsychotics and neuroleptics
T436	Intoxication	12	1	Psychostimulants
T439	Intoxication	8	1	Unsp psychotropic drug
T460	Intoxication	1	0	Cardi-stim glycos/drug simlar act
T510	Intoxication	1	0	Toxic effect of ethanol
T511	Intoxication	1	0	Toxic effect of methanol
T512	Intoxication	0	0	Toxic effect of 2-Propanol
T519	Intoxication	1	0	Toxic effect of unspecified alcohol
T523	Intoxication	0	0	Toxic effects of glycols

T549	Intoxication	1	0	Toxic effects of unspecified corrosive substance
T589	Intoxication	0	1	Toxic effect of carbon monoxide from unspecified source
T599	Intoxication	3	0	Toxic effect of unspecified gases, fumes and vapors
T630	Intoxication	2	0	Toxic effect of snake venom
T659	Intoxication	43	4	Toxic effect of unspecified substance
Y423	Intoxication	3	1	Insulin and oral hypoglycaemic [antidiabetic] drugs
Y433	Intoxication	1	0	Other antineoplastic drugs
Y445	Intoxication	1	0	Thrombolytic drugs
Y450	Intoxication	5	0	Opioids and related analgesics
Y479	Intoxication	4	0	Sedative, hypnotic and antianxiety drug, unspecified
Y575	Intoxication	2	0	X-ray contrast media
Z036	Intoxication	62	1	Encntr for obs for susp toxic eff from ingest sub ruled out
I850	Liver failure	24	1	Esophageal varices
I859	Liver failure	1	0	Secondary esophageal varices with bleeding
K703	Liver failure	1	0	Alcoholic cirrhosis of liver
K704	Liver failure	2	0	Alcoholic hepatic failure
K709	Liver failure	0	0	Alcoholic liver disease, unspecified
K719	Liver failure	1	0	Toxic liver disease, unspecified
K720	Liver failure	7	0	Acute and subacute hepatic failure
K721	Liver failure	1	0	Chronic hepatic failure
K729	Liver failure	247	3	Hepatic failure, unspecified
K746	Liver failure	1	0	Other and unspecified cirrhosis of liver
K750	Liver failure	0	1	Abscess of liver
K754	Liver failure	1	0	Autoimmune hepatitis
K762	Liver failure	1	0	Central hemorrhagic necrosis of liver
K767	Liver failure	1	0	Hepatorenal syndrome
K768	Liver failure	1	0	Other specified diseases of liver
Z944	Liver failure	3	0	Liver transplant status
C019	Malignancy	1	0	Malignant neoplasm of base of tongue
C022	Malignancy	1	0	Malignant neoplasm of ventral surface of tongue
C031	Malignancy	2	0	Malignant neoplasm of lower gum
C068	Malignancy	1	0	Malignant neoplasm of ovrlp sites of and unsp parts of mouth
C139	Malignancy	1	0	Malignant neoplasm of hypopharynx, unspecified
C154	Malignancy	2	0	Malignant neoplasm of middle third of esophagus
C155	Malignancy	3	0	Malignant neoplasm of lower third of esophagus
C159	Malignancy	3	0	Malignant neoplasm of esophagus, unspecified
C169	Malignancy	1	0	Malignant neoplasm of stomach, unspecified
C220	Malignancy	1	0	Liver cell carcinoma
C229	Malignancy	2	0	Malig neoplasm of liver, not specified as primary or sec
C239	Malignancy	1	0	Malignant neoplasm of gallbladder

C240	Malignancy	1	0	Malignant neoplasm of extrahepatic bile duct
C250	Malignancy	4	0	Malignant neoplasm of head of pancreas
C252	Malignancy	0	0	Malignant neoplasm of tail of pancreas
C259	Malignancy	4	0	Malignant neoplasm of pancreas, unspecified
C328	Malignancy	1	0	Malignant neoplasm of overlapping sites of larynx
C329	Malignancy	1	0	Malignant neoplasm of larynx, unspecified
C339	Malignancy	1	0	Malignant neoplasm of trachea
C348	Malignancy	1	0	Malignant neoplasm of overlapping sites of bronchus and lung
C383	Malignancy	1	0	Malignant neoplasm of mediastinum, part unspecified
C410	Malignancy	1	0	Malignant neoplasm of bones of skull and face
C412	Malignancy	1	0	Malignant neoplasm of vertebral column
C443	Malignancy	1	0	Oth and unsp malignant neoplasm skin/ and unsp parts of face
C449	Malignancy	2	0	Other and unsp malignant neoplasm of skin, unspecified
C480	Malignancy	1	0	Malignant neoplasm of retroperitoneum
C499	Malignancy	1	0	Malignant neoplasm of connective and soft tissue, unsp
C559	Malignancy	2	0	Malignant neoplasm of uterus, part unspecified
C569	Malignancy	2	0	Malignant neoplasm of unspecified ovary
C649	Malignancy	1	0	Malignant neoplasm of unsp kidney, except renal pelvis
C729	Malignancy	6	0	Malignant neoplasm of central nervous system, unspecified
C762	Malignancy	1	0	Malignant neoplasm of endocrine gland, unspecified
C770	Malignancy	1	0	Malignant neoplasm of endocrine gland, unspecified
C787	Malignancy	6	0	Malignant neoplasm of endocrine gland, unspecified
C809	Malignancy	158	0	Malignant neoplasm associated with transplanted organ
D339	Malignancy	1	0	Benign neoplasm of central nervous system, unspecified
D361	Malignancy	1	0	Benign neoplasm of prph nerves and autonomic nervous sys
D369	Malignancy	2	0	Benign neoplasm, unspecified site
D391	Malignancy	1	0	Benign neoplasm, unspecified site
D439	Malignancy	5	0	Neoplasm of uncertain behavior of cnsl, unsp
D487	Malignancy	1	0	Neoplasm of uncertain behavior of other specified sites
D489	Malignancy	13	0	Neoplasm of uncertain behavior, unspecified
Z859	Malignancy	2	0	Personal history of malignant neoplasm, unspecified
M419	Musculoskeletal disease	4	0	Scoliosis, unspecified
M480	Musculoskeletal disease	1	0	Spinal stenosis
M539	Musculoskeletal disease	1	0	Dorsopathy, unspecified
F058	Neurological disease	0	1	Delirium due to known physiological condition
F059	Neurological disease	4	2	Delirium due to known physiological condition
G099	Neurological disease	1	0	Sequelae of inflammatory diseases of central nervous system
G122	Neurological disease	5	0	Motor neuron disease
G359	Neurological disease	1	0	Multiple sclerosis
G406	Neurological disease	1	0	Generalized idiopathic epilepsy, intractable, w/o stat epi

G409	Neurological disease	5	1	Epilepsy, unspecified
G419	Neurological disease	32	0	Epilepsy, unsp, intractable, without status epilepticus
G610	Neurological disease	3	0	Guillain-Barre syndrome
G629	Neurological disease	1	0	Polyneuropathy, unspecified
G700	Neurological disease	5	0	Myasthenia gravis
G719	Neurological disease	1	0	Primary disorder of muscle, unspecified
G825	Neurological disease	1	0	Quadriplegia
G839	Neurological disease	0	1	Paralytic syndrome, unspecified
G919	Neurological disease	1	0	Hydrocephalus, unspecified
G934	Neurological disease	1	0	Other and unspecified encephalopathy
G938	Neurological disease	7	0	Other specified disorders of brain
G948	Neurological disease	1	0	Other disorders of brain in diseases classified elsewhere
G969	Neurological disease	1	0	Disorder of central nervous system, unspecified
G989	Neurological disease	6	2	Other disorders of nervous system
G998	Neurological disease	1	0	Oth disrd of nervous system in diseases classified elsewhere
P909	Neurological disease	1	0	Convulsions of newborn
Q079	Neurological disease	1	0	Congenital malformation of nervous system, unspecified
R400	Neurological disease	37	1	Somnolence
R402	Neurological disease	133	6	Coma
R410	Neurological disease	14	2	Disorientation, unspecified
R559	Neurological disease	5	0	Syncope and collapse
R568	Neurological disease	163	4	Post traumatic seizures
R509	Other	1	0	Fever, unspecified
R600	Other	1	0	Localized edema
T670	Other	1	0	Heatstroke and sunstroke
T689	Other	18	0	Hypothermia
Y832	Other	9	0	Anastomos,bypass or grft cause abn react/compl, w/o misadvnt
Z038	Other	1	0	Encntr for obs for oth suspected diseases and cond ruled out
Z039	Other	2	0	Encntr for obs for oth suspected diseases and cond ruled out
K800	Pancreatitis/cholangitis	1	0	Calculus of gallbladder with acute cholecystitis
K810	Pancreatitis/cholangitis	2	1	Acute cholecystitis
K819	Pancreatitis/cholangitis	8	1	Cholecystitis, unspecified
K830	Pancreatitis/cholangitis	11	0	Cholangitis
K832	Pancreatitis/cholangitis	1	0	Perforation of bile duct
K858	Pancreatitis/cholangitis	3	0	Other acute pancreatitis
K859	Pancreatitis/cholangitis	51	8	Acute pancreatitis, unspecified
K862	Pancreatitis/cholangitis	1	0	Cyst of pancreas
K871	Pancreatitis/cholangitis	1	0	Disord of GB, biliary trac and pancreas in dis classd elswhr
R520	Postoperative care, not specified	1	0	Pain, unspecified
R521	Postoperative care, not specified	1	0	Pain, unspecified

R529	Postoperative care, not specified	11	0	Pain, unspecified
Z049	Postoperative care, not specified	376	5	Encounter for examination and observation for unsp reason
F239	Psyciatric disease	1	0	Brief psychotic disorder
F299	Psyciatric disease	3	0	Unsp psychosis not due to a substance or known physiol cond
F329	Psyciatric disease	6	0	Major depressive disorder, single episode, unspecified
F412	Psyciatric disease	1	0	Generalized anxiety disorder
N109	Renal/urologic disease	19	2	Acute tubulo-interstitial nephritis
N111	Renal/urologic disease	1	0	Chronic obstructive pyelonephritis
N133	Renal/urologic disease	1	0	Other and unspecified hydronephrosis
N170	Renal/urologic disease	1	0	Acute kidney failure with tubular necrosis
N178	Renal/urologic disease	2	1	Other acute kidney failure
N179	Renal/urologic disease	74	10	Acute kidney failure, unspecified
N184	Renal/urologic disease	1	0	Chronic kidney disease, stage 4 (severe)
N189	Renal/urologic disease	2	0	Chronic kidney disease, unspecified
N199	Renal/urologic disease	3	0	Unspecified kidney failure
N200	Renal/urologic disease	1	0	Calculus of kidney
N201	Renal/urologic disease	0	1	Calculus of ureter
N209	Renal/urologic disease	1	0	Urinary calculus, unspecified
N999	Renal/urologic disease	1	0	Oth postprocedural complications and disorders of GU sys
Q623	Renal/urologic disease	1	1	Other obstructive defects of renal pelvis and ureter
J380	Respiratory tract disease, except infection or COPD/asthma	1	0	Paralysis of vocal cords and larynx
J384	Respiratory tract disease, except infection or COPD/asthma	29	0	Edema of larynx
J387	Respiratory tract disease, except infection or COPD/asthma	1	0	Other diseases of larynx
J398	Respiratory tract disease, except infection or COPD/asthma	1	0	Other specified diseases of upper respiratory tract
J399	Respiratory tract disease, except infection or COPD/asthma	2	0	Disease of upper respiratory tract, unspecified
J950	Respiratory tract disease, except infection or COPD/asthma	7	0	Tracheostomy complications
P271	Respiratory tract disease, except infection or COPD/asthma	1	0	Bronchopulmonary dysplasia origin in the perinatal period
P288	Respiratory tract disease, except infection or COPD/asthma	1	0	Other specified respiratory conditions of newborn
R042	Respiratory tract disease, except infection or COPD/asthma	0	1	Hemoptysis
R048	Respiratory tract disease, except infection or COPD/asthma	1	0	Hemorrhage from other sites in respiratory passages
R049	Respiratory tract disease, except infection or COPD/asthma	17	0	Hemorrhage from respiratory passages, unspecified
R061	Respiratory tract disease, except infection or COPD/asthma	40	0	Stridor
R068	Respiratory tract disease, except infection or COPD/asthma	1	0	Other abnormalities of breathing
T175	Respiratory tract disease, except infection or COPD/asthma	1	0	Foreign body in bronchus
T179	Respiratory tract disease, except infection or COPD/asthma	17	1	Foreign body in respiratory tract, part unspecified
T189	Respiratory tract disease, except infection or COPD/asthma	2	1	Foreign body of alimentary tract, part unspecified
T273	Respiratory tract disease, except infection or COPD/asthma	6	3	Burn of respiratory tract, part unspecified
J039	Respiratory tract infection	2	0	Acute tonsillitis, unspecified
J042	Respiratory tract infection	16	0	Acute laryngotracheitis
J051	Respiratory tract infection	31	0	Acute epiglottitis

J069	Respiratory tract infection	2	0	Acute upper respiratory infection, unspecified
J099	Respiratory tract infection	2	0	Influenza due to certain identified influenza viruses
J100	Respiratory tract infection	4	3	Influenza due to oth identified influenza virus w pneumonia
J101	Respiratory tract infection	0	1	Flu due to oth ident influenza virus w oth resp manifest
J121	Respiratory tract infection	2	0	Respiratory syncytial virus pneumonia
J129	Respiratory tract infection	2	3	Viral pneumonia, unspecified
J139	Respiratory tract infection	1	2	Pneumonia due to Streptococcus pneumoniae
J152	Respiratory tract infection	0	0	Pneumonia due to staphylococcus
J154	Respiratory tract infection	0	1	Pneumonia due to other streptococci
J155	Respiratory tract infection	1	0	Pneumonia due to Escherichia coli
J159	Respiratory tract infection	167	44	Unspecified bacterial pneumonia
J168	Respiratory tract infection	0	2	Pneumonia due to other specified infectious organisms
J189	Respiratory tract infection	9	4	Pneumonia, unspecified organism
J229	Respiratory tract infection	3	1	Unspecified acute lower respiratory infection
J369	Respiratory tract infection	1	1	Peritonsillar abscess
J390	Respiratory tract infection	3	0	Retropharyngeal and parapharyngeal abscess
J690	Respiratory tract infection	70	6	Pneumonitis due to inhalation of food and vomit
J851	Respiratory tract infection	1	0	Abscess of lung with pneumonia
J869	Respiratory tract infection	4	1	Pyothorax without fistula
J909	Respiratory tract infection	3	0	Pleural effusion, not elsewhere classified
J958	Respiratory tract infection	1	0	Oth intraop and postproc comp and disorders of resp sys, NEC
K122	Respiratory tract infection	36	0	Cellulitis and abscess of mouth
I609	Subarachnoid hemorrhage	78	0	Nontraumatic subarachnoid hemorrhage, unspecified
T809	Surgical and medical complications	1	0	Unsp comp fol infusion, transfuse and theraputc injection
T810	Surgical and medical complications	82	5	Complications of procedures, not elsewhere classified
T812	Surgical and medical complications	6	1	Other postprocedural shock, sequela
T813	Surgical and medical complications	2	0	Disruption of wound, not elsewhere classified
T819	Surgical and medical complications	5	1	Unspecified complication of procedure
T850	Surgical and medical complications	3	0	Mechanical complication of ventricular intracranial shunt
T884	Surgical and medical complications	2	0	Failed or difficult intubation
T885	Surgical and medical complications	1	0	Other complications of anesthesia
T886	Surgical and medical complications	1	0	Anaphylactic reaction due to advrs eff drug/med prop admin
T887	Surgical and medical complications	3	0	Unspecified adverse effect of drug or medicament
T889	Surgical and medical complications	40	0	Complication of surgical and medical care, unspecified
Y699	Surgical and medical complications	6	0	Unspecified misadventure during surgical and medical care
Z948	Transplantation	1	0	Other transplanted organ and tissue status
Z949	Transplantation	349	0	Transplanted organ and tissue status, unspecified
Z988	Transplantation	1	0	Other specified postprocedural states
S220	Trauma	5	0	Fracture of thoracic vertebra
S221	Trauma	0	0	Unspecified fracture of T11-T12 vertebra, sequela

S222	Trauma	1	0	Fracture of sternum
S224	Trauma	11	1	Multiple fractures of ribs
S241	Trauma	6	1	Other and unspecified injuries of thoracic spinal cord
S259	Trauma	3	0	Injury of unspecified blood vessel of thorax
S260	Trauma	1	0	Injury of heart with hemopericardium
S269	Trauma	1	0	Injury of heart, unspecified with or without hemopericardium
S270	Trauma	16	1	Traumatic pneumothorax
S271	Trauma	8	0	Traumatic hemothorax
S272	Trauma	1	0	Traumatic hemopneumothorax
S273	Trauma	2	0	Other and unspecified injuries of lung
S311	Trauma	0	1	Open wound of abdominal wall w/o penetration into perit cav
S328	Trauma	10	0	Fracture of other parts of pelvis
S341	Trauma	1	0	Other and unsp injury of lumbar and sacral spinal cord
S359	Trauma	6	0	Inj unsp blood vess at abdomen, low back and pelvis level
S360	Trauma	1	0	Injury of spleen
S369	Trauma	16	0	Injury of unspecified intra-abdominal organ
S379	Trauma	1	0	Injury of unspecified urinary and pelvic organ
T021	Trauma	0	0	Unspecified injury of unspecified foot, sequela
T079	Trauma	435	66	Unspecified multiple injuries
T093	Trauma	1	0	Unspecified multiple injuries
T109	Trauma	1	1	Unspecified multiple injuries
T119	Trauma	1	0	Unspecified multiple injuries
T129	Trauma	6	2	Unspecified multiple injuries
T139	Trauma	5	0	Unspecified multiple injuries
T149	Trauma	15	0	Unspecified injury
T300	Trauma	21	0	Burn of unspecified body region, unspecified degree
T302	Trauma	1	0	Burn of unspecified body region, unspecified degree
T303	Trauma	1	0	Burn of unspecified body region, unspecified degree
T749	Trauma	8	0	Unspecified maltreatment, confirmed
T754	Trauma	1	0	Electrocution
T792	Trauma	10	0	Traumatic secondary and recurrent hemorrhage and seroma
T794	Trauma	1	0	Traumatic shock
T796	Trauma	3	0	Traumatic ischemia of muscle
T797	Trauma	1	0	Traumatic subcutaneous emphysema
T799	Trauma	1	0	Traumatic subcutaneous emphysema, sequela
G935	Traumatic brain injury	14	0	Compression of brain
G936	Traumatic brain injury	2	0	Cerebral edema
G952	Traumatic brain injury	9	1	Other and unspecified cord compression
G959	Traumatic brain injury	3	0	Disease of spinal cord, unspecified
S019	Traumatic brain injury	6	0	Open wound of unspecified part of head

S021	Traumatic brain injury	2	0	Fracture of base of skull
S027	Traumatic brain injury	1	0	Fracture of mandible of other specified site, sequela
S029	Traumatic brain injury	11	1	Fracture of unspecified skull and facial bones
S060	Traumatic brain injury	8	0	Concussion
S062	Traumatic brain injury	0	0	Diffuse traumatic brain injury
S063	Traumatic brain injury	2	0	Focal traumatic brain injury
S064	Traumatic brain injury	10	1	Epidural hemorrhage
S065	Traumatic brain injury	51	2	Traumatic subdural hemorrhage
S066	Traumatic brain injury	10	0	Traumatic subarachnoid hemorrhage
S0661	Traumatic brain injury	1	0	Traumatic subarachnoid hemorrhage
S069	Traumatic brain injury	94	4	Unspecified intracranial injury
S099	Traumatic brain injury	1	0	Unspecified injury of face and head
S119	Traumatic brain injury	2	0	Open wound of unspecified part of neck
S1200	Traumatic brain injury	1	0	Unspecified fracture of first cervical vertebra
S121	Traumatic brain injury	1	1	Fracture of second cervical vertebra
S122	Traumatic brain injury	0	1	Fracture of third cervical vertebra
S129	Traumatic brain injury	13	0	Fracture of neck, unspecified
S140	Traumatic brain injury	1	0	Concussion and edema of cervical spinal cord
S141	Traumatic brain injury	15	1	Other and unspecified injuries of cervical spinal cord
S159	Traumatic brain injury	4	0	Injury of unspecified blood vessel at neck level
